# Tonsillectomy remains a questionable option for pediatric autoimmune neuropsychiatric disorders associated with streptococcal infections (PANDAS)

**DOI:** 10.3205/cto000134

**Published:** 2016-12-15

**Authors:** Jochen P. Windfuhr

**Affiliations:** 1 Department of Otolaryngology, Head & Neck Surgery, Allergology, Kliniken Maria Hilf, Mönchengladbach, Germany

**Keywords:** Tonsillectomy, PANDAS, Sydenham’s chorea, rheumatic fever, Streptococci, tics, obsessive compulsive disorders

## Abstract

**Background:** Pediatric autoimmune neuropsychiatric disorders associated with streptococcal infections (PANDAS) is a disease attributed to children with obsessive compulsive disorders (OCD) or tic disorders associated with streptococcal infections. Because otolaryngologists examine a large number of pediatric patients with recurrent streptococcal infections, tonsillectomy (TE) is a common option of therapy. This study was conducted to evaluate the efficacy of TE in patients presenting with verified PANDAS.

**Material and methods:** A PubMed review was performed using search terms “tonsillectomy” and “PANDAS”, “OCD”, “compulsive” “pediatric autoimmune”, “chorea” and “tic” limited by publication date of January 1, 1995, to July 31, 2015. Reviews without patients were not included in the review.

**Results:** Nine papers matched our search criteria, including 6 case reports with 8 patients and 3 case series. Most case reports were in favor of TE, but this was by far not supported by the findings in the case series. The follow-up ranged from 2 to 36 months in case reports and from 24 to 36 in case series.

**Conclusion:** Establishing the diagnosis of PANDAS is complicated because of underlying comorbidities in the field of neurology-psychiatry and the lack of a reliable biomarker. The positive outcome after TE as reported in case studies may be influenced by the postoperative medication and is not supported by the results of large-scale studies. In the light of the considerable postoperative morbidity rate, it appears wise to indicate TE for PANDAS only in supervised clinical studies.

## 1 Introduction

For the first time in 1998, Swedo et al. from the National Institute of Mental Health in Maryland reported of 50 children with obsessive compulsive disorders (OCD) and/or tic disorders that were associated with an group A β-hemolytic streptococci (GABHS) infection and deteriorated from episode to episode [1]. Three years earlier a first case report of this observation had been published [2]. Swedo et al. assumed an independent disease which they labeled *Pediatric Autoimmune Neuropsychiatric Disorders Associated with Streptococcal Infections* (PANDAS). According to their hypothesis, symptoms develop abruptly and 3 years earlier than without streptococcal infections. Moreover, this entity is characterized by conspicuities such as separation anxiety, emotional instability, or attention deficit hyperactivity syndrome. Male gender and a family history were identified as risk factors in the case series [3]. According to the authors, 5 clinical criteria characterize the entity:

1. presence of OCD and/or tic disorder
2. prepubertal symptom onset
3. sudden onset or abrupt exacerbations (sawtooth course)
4. association with neurological abnormalities (presence of adventitious movements or motoric hyperactivity during exacerbations), and
5. temporal association between symptom exacerbations and GABHS infections.

Repeated episodes of GABHS-associated tonsillitis despite adequate antibiotic therapy are a common indication for tonsillectomy (TE) [4]. Stimulated by a clinical case, we analyzed the scientific literature to clarify, whether or not PANDAS patients may also benefit from TE. 

## 2 Material and methods

### 2.1 Case report

A 9-year-old girl with a suspected PANDAS syndrome was presented to verify the indication for TE. The parents reported monthly recurring fever attacks associated with tonsillitis, and also a variety of neurological symptoms. All findings on the day of examination were inconspicuous, the tonsils were very small, did not show any detritus, and were not reddened. The entire pharyngeal mucosa was normal. Surgical interventions were denied in the girl’s history, the delivery was timely and the child’s development had been normal until two years ago.

At that time, the girl fell sick of meningoencephalitis after a camping holiday, the origin of the disease could never be identified. She had been hospitalized for two weeks. At the beginning, she received vancomycin and ceftriaxone but because of a deterioration of her condition (fever in the evening of more than 40°C for 5 days, seizures for 3 minutes, altered EEG, increasingly reduced vigilance) the medication was changed to doxyclycline and prednisone. This resulted in an improvement, the fever disappeared and she fully recovered within a few months. 

Analysis of the liquor could not reveal any bacteria but increased leukocyte values. After three weeks, the therapy was stopped and the child remained free of infections for 18 months. After a third episode of sore throat with swelling of the cervical lymph nodes, the diagnosis of PANDAS was made because she started crying routinely during school without any reason or because of vanities and sudden onset of OCD. Furthermore, she had become photophobic, phonophobic, and touch-sensitive. In addition, she had developed arithmomania (counting tiles in the hall) and fears especially of severe diseases and negative thoughts. All symptoms had disappeared regularly under antibiotic therapy. Because of the poor evidence, tonsillectomy was not recommended. And because of the small size of the tonsils, even tonsillotomy was not indicated by the author.

Later, the parents reported that only in about one third of the repeatedly performed swabs, streptococci were identified. Furthermore, the fever occurring in intervals of three weeks was accompanied by headaches, vertigo, and swellings of the cervical lymph nodes as well as blue-black spots on the legs, sometimes pain in the legs, and red nodules of the skin. All symptoms always responded to antibiotic therapy, however, the number of blood platelets remained constantly low (106,000/µl). The suspicion of Bartonella infection was confirmed by serology and is now treated with antibiotics. After consultation with additional physicians, there is a hypothesis that in addition to bartonella, there may be a concurrent staphylococcus, Borrelia or protozoal infection and she is now being treated for those as well. The parents reported that in between febrile episodes she is as happy, healthy, and normal as she ever was, except for some intermittent fatigue. She has another appointment with an ENT coming up, in addition to the recent addition of some anti-protozoal medications.

The author was informed by November 2016, that meanwhile the girl had undergone tonsillectomy and remained free of symptoms since then (60 days). The parents were informed, that resistant staphylococcus and pseudonomas were identified in her tonsils.

### 2.2 Terminology

#### 2.2.1 Obsessive compulsive disorders (OCD)

The term of OCD compulsive disorders encompasses obsession, rumination, compulsion, and compulsive rituals [5]. Obsessions are thoughts, imaginations, or impulses that recur and persist despite efforts to ignore or confront them. They are nearly always torturing and patients often try in vain to get rid of them. The thoughts are experienced as belonging to the own personality even if the patients perceive them as involuntary and often disgusting. Compulsions or compulsive rituals are stereotypes that are continuously repeated. They are neither perceived as comfortable nor do they contribute to fulfill a useful task. The patients often experience them as prophylaxis against an objectively improbably event that might harm them or make them cause harm to others. In general, such a behavior is experienced as useless and ineffective and patients try hard to fight it. In most cases, anxiety is present. If compulsions are suppressed, anxiety significantly increases. About 2–4% of children and adolescents are affected by OCD, probably as a consequence of a damage of the basal ganglia with genetic predisposition and disorders of the immune modulation [3], [6].

#### 2.2.2 Tics

A tic is an involuntary, repetitive, non-rhythmic motor movement or vocalization involving discrete muscle groups that starts suddenly and does not pursue any purpose [7]. Usually, tics are experienced as impossible to be voluntarily influenced but they can mostly be suppressed for intervals of different lengths. Stress may enhance them, during sleep they disappear. Frequently observed motor tics are eye blinking, head jerking, shoulder shrugging, and facial grimacing. Simple phonic tics are for example throat clearing, sniffing, grunting, and hissing. Complex tics may show as self-beating, jumping, and hopping. Symptoms of complex vocal tics encompass repeating of certain words and sometimes using socially inadequate and even obscene words (coprolalia) and repeating one’s own previously spoken sounds or words (palilalia). Transient tic disorders consist of multiple motor tics, phonic tics or both, with a duration of 1 to 12 months. Frequent tics are eye blinking, facial grimacing, or head jerking. Chronic tic disorder is by definition a motor or phonic tic (but not both) lasting for at least one year. Diagnosis of Tourette's syndrome is based on multiple motor tics, and at least one phonic tic, not necessarily occurring at the same time. The disorder deteriorates usually in adolescence and is likely to persist until adulthood. Vocal tics are often multiple with explosive repetitive vocalizations, throat clearing, grunting, and the use of obscene words or phrases. Sometimes concomitant echopraxia is observed that may also be of obscene nature (copropraxia). 

#### 2.2.3 Chorea minor (Sydenham’s chorea)

Sydenham’s chorea is acknowledged as one of the main Jones criteria of acute rheumatic fever, such as carditis, polyarthritis, erythema anulare, and subcutaneous nodes [8]. Minor criteria are arthralgia, fever, pathological laboratory parameters (ESR, C-reactive protein) and prolonged PQ interval [9]. The symptoms include hyperkinesia of the hands, the throat, the facial muscles, and muscular hypotonia and hyporeflexia at the same time. It is resulting from an auto-antibody-induced damage of the basalganglia (striatum) [9].

### 2.3 Review of the literature

Studies were searched in the PubMed database entering the key words of *tonsillectomy* and *PANDAS*, *OCD*, *compulsive*, *pediatric autoimmune*, *chorea*, and *tic* where tonsillectomy was performed to treat PANDAS. Articles were included if published between 1995 and July 31, 2015. Review articles on PANDAS without presentation of patients’ histories were excluded.

## 3 Results

Only 8 of 18 articles matched our search criteria and were eligible for further analysis [6], [10], [11], [12], [13], [14], [15], [16]. One article had been cited and was also included [17]. The articles had been published between 2001 and 2015, 5 of them originated from US American groups (Table 1 (Tab. 1)).

Six articles reported of a benefit after TE occurring in 8 patients with a presurgical neurological disorder [6], [12], [13], [14], [15], [17]. 7 of these 8 patients were male and there were two pairs of siblings among the patients [6], [15]. In one case, TE had been indicated exclusively because of upper airway obstruction resulting from tonsillar hyperplasia, the effect was considered as coincidental and unintended [17]. In addition to the case reports we identified 3 case series with a total of 173 patients of whom 91 had undergone TE. 95 of these 173 children were male (54.9%).

Data on the anti-streptolysin titer were provided for 5 patients, the anti-DNase titer was determined in 2 patients of the case reports. In the case series, only one of 3 publications did not measure the anti-streptococci titer. The follow-up varied between 2 and 36 months for the case reports (not stated for one case). The follow-up was not stated in one of the case series, and the patients were followed for 2 years and at least 3 years, respectively.

Complete therapeutic success was reported in 4 [6], [12], [14], [17] of the 8 single cases and in one case series [10] for further 3 patients. A benefit was not verified for the total of 82 patients who had undergone TE in the other two case series [11], [16], not even a positive tendency. 

## 4 Discussion

### 4.1 Diagnostics

We experienced in our case, that TE for PANDAS is also discussed by non-medicals, preferably in the social media. It should be noted that description of the symptoms may be biased by non-scientific interpretation and wishful thinking of patients/parents. We therefore aimed to collect scientific data from the PubMed database as an attempt of a scientific reply.

As early as in 1894, Osler suggested a clinical correlation between Sydenham’s chorea caused by a lesion of the basal ganglia resulting from a streptococcal infection. The theory of Swedo’s working group, that GABHS infection might also trigger psychiatric disorder needs to be proven [1]. The working group of Swedo based their statements either on smear tests and/or antibody measurements against streptococci. Further details are not obtainable from the paper. The authors admit that a temporary correlation was subject to variations with possibly even missing proof of streptococci. Even in the context of Sydenham’s chorea this problem might occur due to the fact of a symptom delay of 6 to 9 months after the infection. It should be emphasized that diagnosis of a streptococcal infection was accepted not only by an adequate medical documentation and prospective measurements but also if reported by the parents. This must be seen very critical because in our case the girl’s parents relativized the initial statements and finally reported a positive result for approximately half of the episodes. In 21 of 50 children the test was positive at the onset; one child was exposed to GABHS and 14 developed symptoms after pharyngitis without GABHS detection. A proven GABHS infection at least 6 weeks before symptom exacerbation was diagnosed in every child. In total, 144 GABHS-associated exacerbations were registered, but only in 45 cases the proof was made by swabs or alternatively scarlet fever was diagnosed. Streptococcal infection proven only once is considered as insufficient and repeated tests are recommended [1]. According to Mell et al., streptococcal infection represents a clear risk factor for the development of OCD or tic with a disposition that is still not clarified [18].

In cases of OCD and tics, also MRI-radiological changes of the basal ganglia as well as antineuronal antibody blood values could be identified beside the clinical signs [6], [19]. The single case reports show that despite clear PANDAS symptoms no conspicuities could be seen in the MRI examinations [13], [14]. Interestingly, an increased anti-streptococci titer was identified in Tourette’s syndrome, which is a subtype of tic disorder. The level correlated with the severity of the symptoms [20]. According to Peterson et al., those results are caused by a simultaneous attention deficit hyperactivity syndrome and not by the OCD or tic [21]. A close timely relation between the titer and the severity of the disease, however, could not be confirmed for children with PANDAS. High titers do not confirm the diagnosis, neither does a negative result exclude the diagnosis, and furthermore high levels may even persist after TE [15], [16], [17], [22]. Thus the value of this investigation for the diagnosis and follow-up of PANDAS appears questionable. Another criticism aims at the use of antibody titers without taking into account the patients’ age, which was neglected in every publication. Moreover, the impact of co-factors on laboratory parameters by the synchronous neurological basic disease was not analyzed in any of the case series. 

Hints that increased titers of the monoclonal antibody D8/17 against surface antigens of B lymphocytes in rheumatic fever were predisposing for the development of psychiatric disorders in children [3] were not confirmed by Inoff-Germain et al. [23]. This approach was not further pursued in the case reports or case series. In the trial of Walls et al. from 2015, molecular genetic examinations of tonsillar tissue of 12 PANDAS patients were performed and compared with the results of 6 children undergoing TE to resolve either upper airway obstruction or recurrent episodes tonsillitis. Among the different chemokines and cytokines, significant differences were found for eotaxin-3, interleukin-8, interferon-inducible protein 10, interferon-γ, interleukin-10, and tumor necrosis factor α. The authors legitimately state that the value of these findings for clinical decisions has to be clarified in future studies [24]. 

### 4.2 High familial prevalence

OCD and tics seem to occur with higher familial incidences [1], [6]. In the present study, this was reported only by Orvidas and Slattery as well as Heubi and Shott for two pairs of siblings in each case report [6], [15]. In their publication, Arostegui et al. reported of a 5-year-old girl whose mother had a history of OCD [25]. Familiarity was not registered in any of the other publications.

### 4.3 Therapy

A first report about TE as therapeutic option for children with PANDAS was published in 2001 by Orvidas and Slattery [6]. At that time, the intervention was not mentioned as therapeutic option by Arnold and Richter. Instead, serotonin antagonists and behavioral therapy were suggested with success rates between 50% and 100%. According to the authors, other therapy types such as plasmapheresis or intravenous application of immunoglobulin [26] had the character of trials, adjuvant antibiotics were recommended only in cases with positive streptococci testing [3]. On several occasions antibiotics had a positive effect on exacerbations when they were associated with infections [14], [27]. This was also reported by the girl’s parents in our case. However, behavioral therapy was not performed in that girl. Only Fusco et al. [13] reported of successful application of tetrabenazine in combination with penicillin as adjuvant therapy of PANDAS, final conclusions, however, cannot be drawn.

The success rate of TE seems to be very high in the light of the 6 case reports because in 4 of the reported 8 cases the children were symptom-free during a follow-up period of at least 11 months. In one case each, the child developed a singular panic attack, centrally effective drugs were reduced and finally stopped after 12 months and one child showed significant improvement (Table 1 (Tab. 1)). Demesh et al. reported a benefit in 3 of 9 children after TE with complete remission and a tendency of a reduced severity in the remainder. The significance of this study, however, is very limited because the data depended on the memory of the parents, neurological conspicuities were evaluated by means of a non-validated procedures, and the data were not compared by a control group [10].

The data analysis of the other 2 case series could not confirm any benefit from TE, neither clinically nor by laboratory values (anti-streptococci titer). A closer look to the publication of Murphy et al. reveals that 10 patients had undergone adenoidectomy, 22 children adenotonsillectomy, and 4 children TE, respectively [11], [16].

### 4.4 Prophylaxis

In the study of Garvey et al., antibiotic therapy was ineffective as prophylaxis [28] in contrast to other reports [22], [27], [29]. Moreover, the prophylactic value of TE appears questionable since in the case series of Murphy et al. 20 of 43 children had already undergone TE before developing PANDAS [16]. The authors reported that TE after onset of PANDAS was not capable to prevent further episodes of the disease. The significance of the trial is very limited since only patients with OCD and/or tic were followed, not those with successful outcome after TE. Unfortunately, the patients were not prospectively followed (neither clinically nor serologically). Therefore, it cannot be excluded that the group evaluated a particular subpopulation. However, TE was without effect on several anti-streptococci titer or the severity of the disease.

Interestingly, a PANDAS was observed 8 days after TE. After application of sertraline/clorazepin as well as amoxicillin for several weeks, this 5-year old girl remained symptom-free until the end of the follow-up period (12 months) [25].

## 5 Summary

Establishing the diagnosis of PANDAS may be difficult due to existing neurologic-psychiatric comorbidities and the lack of reliable biomarkers. Moreover, the benefit of TE is hard to assess when centrally effective medication is continued in patients. This may be obvious in case reports [12], [15] but is not detectable in case series. Large-scale case series could not confirm the benefit of TE for PANDAS that was suggested in case reports. Due to the considerable post-tonsillectomy morbidity it appears wise to recommend the intervention only as a part of a controlled trial with standardized endpoints and ideal surveillance.

## Notes

### Competing interests

The author declares that he has no competing interests.

## Figures and Tables

**Table 1:**
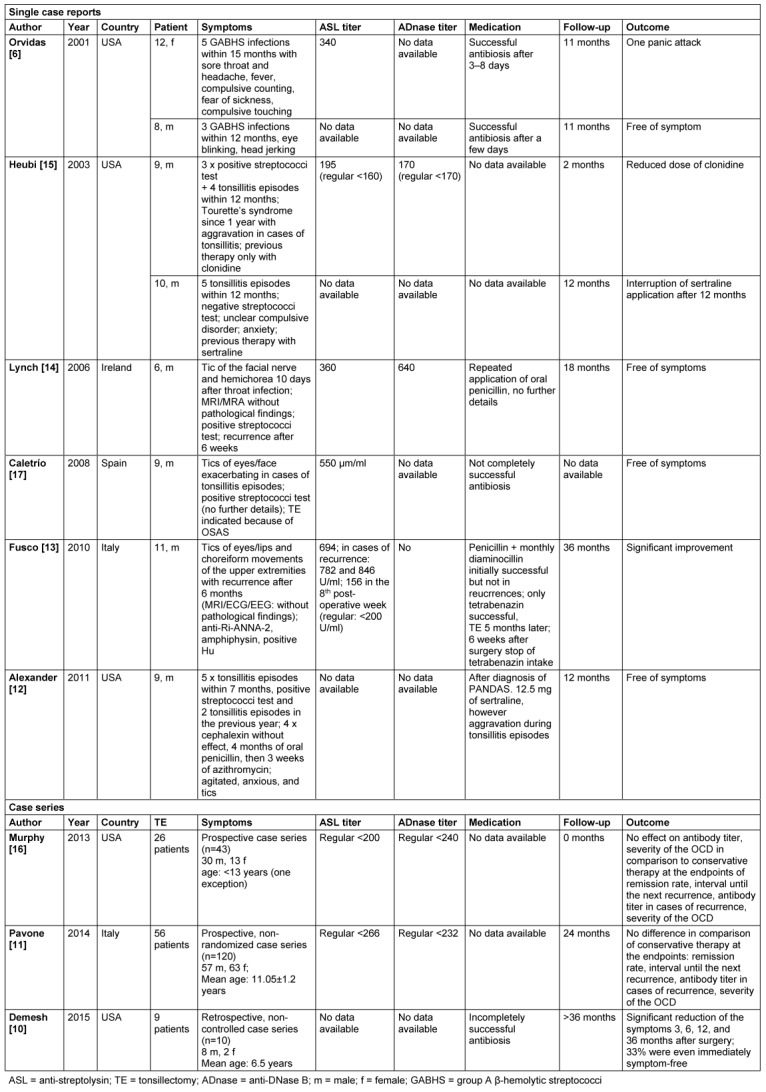
Reports about tonsillectomy and PANDAS
